# Clinical Efficacy and Safety of Different Doses of Sildenafil in the Treatment of Persistent Pulmonary Hypertension of the Newborn: A Network Meta-analysis

**DOI:** 10.3389/fphar.2021.697287

**Published:** 2021-09-24

**Authors:** Linli Sun, Chunxia Wang, Yulu Zhou, Wei Sun, Chunjiang Wang

**Affiliations:** ^1^ Department of General Surgery, The Third Xiangya Hospital, Central South University, Changsha, China; ^2^ Department of Pharmacy, Yinan County People’s Hospital, Linyi, China; ^3^ Department of Pharmacy, The Third Xiangya Hospital, Central South University, Changsha, China

**Keywords:** sildenafil, persistent pulmonary hypertension, newborn, different doses, clinical efficacy

## Abstract

**Objective:** To evaluate the efficacy and safety of different doses of sildenafil for persistent pulmonary hypertension of the newborn (PPHN) with Bayesian random effects network meta-analysis.

**Methods:** We searched Chinese and English databases for randomized controlled trials (RCTs) concerning sildenafil in newborns with persistent pulmonary hypertension from 1998 to December 2020.

**Results:** Twenty-two RCTs including over 2131 patients were included. Sildenafil was administered by nasal feeding at 0.3–2 mg/kg every 4–6 h. The network meta-analysis revealed that 1.5 mg/kg of sildenafil led to a significant decrease in pulmonary artery systolic pressure (PASP) compared with 0.3 and 0.6 mg/kg (*p* < 0.05); 1.5 mg/kg was better than 0.3, 0.5, and 1.0 mg/kg at increasing the partial pressure of oxygen (PaO_2_) (*p* < 0.05); 1.5 mg/kg was better than 0.5, 0.6 and 1.0 mg/kg at reducing the partial pressure of carbon dioxide (PaCO_2_) (*p* < 0.05); and 1.2 mg/kg was better than 0.3, 0.5 and 1.0 mg/kg at increasing the arterial oxygen saturation (SaO_2_) (*p* < 0.05). The surface under the cumulative ranking analysis (SUCRA) results showed that 1.5 mg/kg had the best effect in reducing PASP (SUCRA = 92.0%, moderate certainty evidence) and PaCO_2_ (91.1%) and increasing PaO_2_ (SUCRA = 79.3%, moderate certainty evidence), 2.0 mg/kg had the best effect in increasing SaO_2_ (SUCRA = 88.6%, moderate certainty evidence) and total effective rate (SUCRA = 93.5%, low certainty of evidence)). No severe adverse effects were observed with the different doses of sildenafil.

**Conclusion:** Different doses of sildenafil can significantly improve PPHN, and 1.5 mg/kg of sildenafil has better clinical efficacy and does not increase the probability of adverse reactions.

## Introduction

Persistent pulmonary hypertension of the newborn (PPHN) is one of the most serious fatal diseases in the neonatal period; this condition mostly occurs during the transition from fetal to adult circulation. It is characterized by elevated pulmonary vascular resistance (PVR) that leads to labile hypoxia in the immediate postnatal period with or without respiratory distress (Jain and McNamara, 2015). The incidence of PPHN is approximately 1.8–1.9 per 1000 live births in the United States (Steurer et al., 2017) and 1.2–4.6 per 1000 live births in Asian countries (Nakwan et al., 2020) with an overall mortality rate ranging from 7 to 15% (Clark et al., 2000; Steurer et al., 2017) and 20.6% (Nakwan et al., 2020), respectively. PPHN survivors suffer from long-term sequelae including chronic oxygen dependence, stroke, and cognitive, neurodevelopmental and hearing abnormalities (Sharma et al., 2005).

PPHN remains a major cause of morbidity and mortality in neonatal centers across the globe. It is therefore essential to take the available treatment modalities to ensure better outcomes. Phosphodiesterase (PDE) inhibitors have recently been studied as therapeutic agents for PPHN. Sildenafil is the most well-researched PDE5 inhibitor for PPHN. It was approved for the treatment of pulmonary hypertension (PH) in adults in 2005. Notably, sildenafil is not approved for use in PPHN. To date, most case reports and prospective or retrospective studies (Baquero et al., 2006; Mourani et al., 2009) have shown that sildenafil can be used successfully to improve the oxygenation parameters in neonates with PPHN especially in a resource-limited setting where treatments such as extracorporeal membrane oxygenation (ECMO) and inhaled nitric oxide (iNO) are not available. It was well tolerated and was not associated with short-term side effects.

Unfortunately, pharmacokinetic data on the optimal dose regime in neonates are limited. Sildenafil dosing data vary widely between sources for PPHN, and the indications have been derived from data from adults and older children. To date, there is still considerable controversy about the dosage of sildenafil for PPHN, and an optimal dose has not yet been recommended. The European Medicines Agency (EMA) recommends its use at “low doses.” NeoFax, a well-known neonatal drug manual published in 2006, states that a dose of 0.3–1 mg/kg can be given every 6 h for newborns with PPHN. In 2011, the recommended dose of sildenafil was 0.5–2 mg/kg every 6 h with a maximum dose of 3 mg/kg. The British National Formulary suggested including incremental doses ranging from 0.5 to 2 mg/kg every 6 h (Joint Formulary Committee, 2010-2011). The American Heart Association (AHA) and the American Thoracic Society (ATS) suggest a dose of 0.5–1 mg/kg three times daily orally (Abman et al., 2015). In China, sildenafil is administered orally at a dose of 0.5–1 mg/kg every 6 h for PPHN (The Subspecialty Group of Neonatology and Pediatric Society, Chinese Medical Association, 2017). Recently, a growing number of randomized controlled studies (RCTs) have investigated the efficacy of different doses of sildenafil for PPHN. For this reason, we used a network meta-analysis (NMA) to evaluate the efficacy of different doses of sildenafil for PPNH in order to find the optimal sildenafil dose and provide a decision-making basis for PPHN.

## Methods

### Participants and Research Type

Newborns from 0 to 28 days postpartum who met the diagnostic criteria for PPHN of pulmonary artery systolic pressure (PASP) > 35 mmHg were included. PPHN can be formed by special anatomical and physiological characteristics in the neonatal period, and can also be secondary to parenchymal lung diseases, meconium aspiration syndrome (MAs), respiratory distress syndrome (RDs), pneumonia or sepsis with or without asphyxia (The Subspecialty Group of Neonatology and Pediatric Society, Chinese Medical Association, 2017). All infants were given continuous mechanical ventilation and different doses of sildenafil treatment by oral or nasal feeding without iNO, vasoactive medications, milrinone or ECMO. RCTs were published in English and Chinese. There were no gender restrictions. The review protocol was registered at the Prospero international prospective register of systematic reviews (registration no. CRD42021260652).

### Inclusion and Exclusion Criteria

The inclusion criteria were as follows: *1*) newborns with a confirmed diagnosis of PPHN, *2*) randomized controlled clinical trials, *3*) studies that involved various different doses for PPNH, and *4*) outcome indices including pulmonary artery pressure. Studies were excluded for the following reasons: texts that were not in English or Chinese, duplicate publications, case reports or reviews and texts with no observation indicators.

### Search Strategy and Selection Criteria

We searched the Wanfang, China National Knowledge Infrastructure (CNKI), China Biology Medicine disc (CBMdisc), PubMed, EMBASE, Web of Science and Cochrane Central Register of Controlled Trials databases for RCTs that examined the efficacy of sildenafil for PPHN from 1998 to December 2020 by using “sildenafil,” “PPHN,” “persistent pulmonary hypertension of the newborn,” “pulmonary hypertension,” “Viagra,” “persistent fetal circulation syndrome,” “persistent fetal circulation,” and “newborn.”

### Data Extraction and Quality Assessment

Two independent investigators reviewed the titles, abstracts and full articles that satisfied the inclusion criteria and independently extracted data into a predetermined database. The following information was extracted from each trial: the first author’s name, year of publication, demographic characteristics of participant, sample size, study design, interventions and treatment duration, and observation indicators. We assessed the risk of bias using the Cochrane risk of bias tool version 2 (Cochrane Collaboration, Oxford, England) as low, high, and unclear risk of bias, and any disagreements were resolved by consensus.

### Outcome Measures

If the mean and standard deviation (SD) of the change in scores were not directly extracted from the literature, they were calculated by using the following formula recommended by the Cochrane Handbook for Systematic Reviews of Interventions, where SD_baseline_ and SD_final_ represent the SD at baseline and follow-up, respectively, and Corr represents a correlation coefficient that describes the similarity between baseline and follow-up measurements (Higgins et al., 2021). Here, we choose Corr = 0.5 based on the sensitivity analysis result of Corr value.

$${\text{Mean}}_{\text{change}}={\text{Mean}}_{\text{final}}-{\text{Mean}}_{\text{baseline}}$$

$${\text{SD}}_{\text{change}}=\sqrt{SD_{baseline}^{2}+SD_{final}^{2}-\left(2*\text{Corr}*SD_{baseline}*SD_{final}\right)}$$

A 10% decrease from baseline in PASP or an PASP <35 mmHg was considered effective after the newborn is treated with sildenafil. The calculation method of total effective rate was the proportion of neonates with PASP decreased by 10% or PASP<35 mmHg after sildenafil treatment.

### Statistical Analysis

We conducted to pool the results of direct and indirect comparisons using a Bayesian approach. We will fit our model using STATA 14 statistical software (StataCorp, College Station, TX, United States) and Aggregate Data Drug Information System 1.16.8 software (Drug Information Systems, Groningen, Netherlands). The odds ratio (OR) with a 95% confidence interval (CI) was applied to evaluate the end points. For the NMA, ORs with 95% credible intervals (CrIs) were used. The NMA under the Bayesian framework, using Markov chain Monte Carlo (MCMC) random effects model. A consistency model based on the MCMC simulation method was applied by using 50,000 simulation iterations for each four chains with a burn-in period of the first 20,000 iterations. Node-splitting analysis and inconsistency standard deviation (ISD) were then performed to evaluate the consistency of the data. A two-tailed *p* value of <0.05 was considered to be significant. A value of *I* ^*2*^ = 0–50% was considered as low heterogeneity; 50–75% as moderate heterogeneity; and 75–90% as high heterogeneity. If the difference was not statistically significant, it indicates that the direct and indirect comparison results were consistent, and the consistency model was used for analysis. If there were differences, we used inconsistent model analysis. Surface under the cumulative ranking analysis (SUCRA) was used to rank the effects of the treatment regimens. The SUCRA results are expressed as percentages to compare each intervention to an imaginary intervention, which was always the best intervention without uncertainty. Throughout the meta-analysis process, we had followed the Preferred Reporting Project (PRISMA) guidelines for systematic reviews and meta-analysis.

We will also assess the quality of evidence contributing to network estimates of the main outcomes with the GRADE framework, which characterises the quality of a body of evidence on the basis of the study limitations, imprecision, inconsistency, indirectness and publication bias.

## Results

### Characteristics of the Included Studies

**FIGURE 1:**
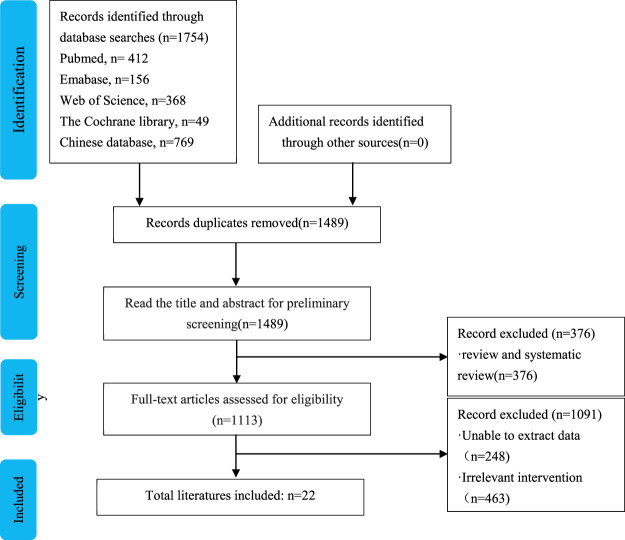
Flow chart of literature screening.

The search strategy identified 1754 studies. Finally, 22 RCTs with over 2,131 patients were included in the analysis (Chen et al., 2012; Li, 2013; Liu and Huang, 2013; Yang et al., 2013; Chen, 2014; Sun and Zhang, 2014; Dong, 2015; Feng, 2015; Li, 2015; Luo et al., 2015; Mo et al., 2015; Tan, 2016; Chen and Wang, 2017; Li and Mo, 2017; Tian, 2017; Wang et al., 2017; Li, 2018; Pan and Wei, 2018; Wang and Li, 2019; Zhao and Bai, 2019; Zhu, 2019; Higgins et al., 2021). The flowchart of the literature retrieval process is shown in Figure 1. The studies were published between 2015 and 2018 and included seven doses: 0.3, 0.5, 0.6, 1.0, 1.2, 1.5, and 2.0 mg/kg. The characteristics of the included RCTs were shown in Table 1. Sildenafil was administered by nasal feeding at 0.3–2 mg/kg every 4–6 h, and the course of treatment was 3–6 days. Eleven studies treated PPHN patients using the brand name drug, seven studies used unspecified manufacturers, and four studies used generic drugs.

**TABLE 1:** Baseline characteristics of the included studies.

Author year	Intervention	Route of administration	Sample size	Frequency	Duration	Outcome indicators
Zhang 2018 (Zhang, 2018)	a/b/d/f	Nasal feeding	40/40/40/40	Q6h	4 days	①②③④
Zhu 2019 (Higgins et al., 2021)	d/g	Nasal feeding	68/68	Q6h	3–6 days	①③
Feng 2015 (Zhu, 2019)	c/d/g	Nasal feeding	20/20/20	Q4-6h	3–6 days	①②③⑤
Li 2018 (Feng, 2015)	b/d/f/g	Nasal feeding	50/50/50/50	Q6h	3 days	①④
Sun 2014 (Li, 2018)	a/c/e/g	Nasal feeding	10/10/10/10	Q6h	4–5 days	①②③④
Zhao 2019 (Sun and Zhang, 2014)	b/d/g	Nasal feeding	40/40/40	Q6h	3 days	①③④⑤
Pan 2018 (Zhao and Bai, 2019)	a/b/d	Nasal feeding	56/56/56	Q6h	3–6 days	①③④⑤
Wang 2019 (Pan and Wei, 2018)	b/d/g	Nasal feeding	26/26/26	Q6h	3 days	①③④⑤
Li 2013 (Wang and Li, 2019)	c/d/g	Nasal feeding	18/18/18	Q4-6h	3 days	①②③④⑥⑦
Li 2017 (Li, 2013)	a/b/d	Nasal feeding	26/26/26	Q6h	3 days	①③④⑤⑥⑦
Chen 2012 (Li and Mo, 2017)	a/b/d	Nasal feeding	18/15/18	Q6h	3 days	①③④⑤⑥⑦
Li 2015 (Chen et al., 2012)	c/e/g	Nasal feeding	24/24/24	Q6h	3–5 days	①③
Mo 2015 (Li, 2015)	d/g	Nasal feeding	50/50	Q6-12h	3 days	①④
Luo 2015 (Mo et al., 2015)	a/b/d	Nasal feeding	50/50/50	Q6h	3 days	①③④⑤⑥⑦
Liu 2013 (Luo et al., 2015)	c/d/g	Nasal feeding	20/20/20	Q4-5h	3 days	①②③⑤
Tian 2017 (Liu and Huang, 2013)	a/c/d	Nasal feeding	80/80/80	Q6h	5 days	①③④⑤
Chen 2017 (Tian, 2017)	b/d/g	Nasal feeding	20/20/20	Q6h	3 days	①②③④⑤
Dong 2015 (Chen and Wang, 2017)	c/d/g	Nasal feeding	6/11/8	Q5h	3–5 days	①②③⑤⑥⑦
Yang 2013 (Dong, 2015)	b/d/g	Nasal feeding	10/10/10	Q6h	5 days	①③④⑤
Wang 2017 (Yang et al., 2013)	b/d	Nasal feeding	40/40	Q8h	3 days	①③④⑤⑥
Chen 2014 (Wang et al., 2017)	c/d/f	Nasal feeding	25/25/25	Q6h	3–5 days	①③⑥⑦
Tan 2016 (Chen, 2014)	b/d/f	Nasal feeding	35/35/35	Q6h	3–5 days	①③⑥⑦

a: 0.3 mg/kg, b: 0.5 mg/kg, c: 0.6 mg/kg, d: 1.0 mg/kg, e: 1.2 mg/kg, f: 1.5 mg/kg, g: 2.0 mg/kg; ① pulmonary artery systolic pressure (PASP), ② systolic blood pressure (SBP), ③ partial pressure of oxygen (PaO_2_), ④ partial pressure of carbon dioxide (PaCO_2_), ⑤ arterial oxygen saturation (SaO_2_) ⑥ adverse reaction, ⑦ clinical efficacy.

**FIGURE 2:**
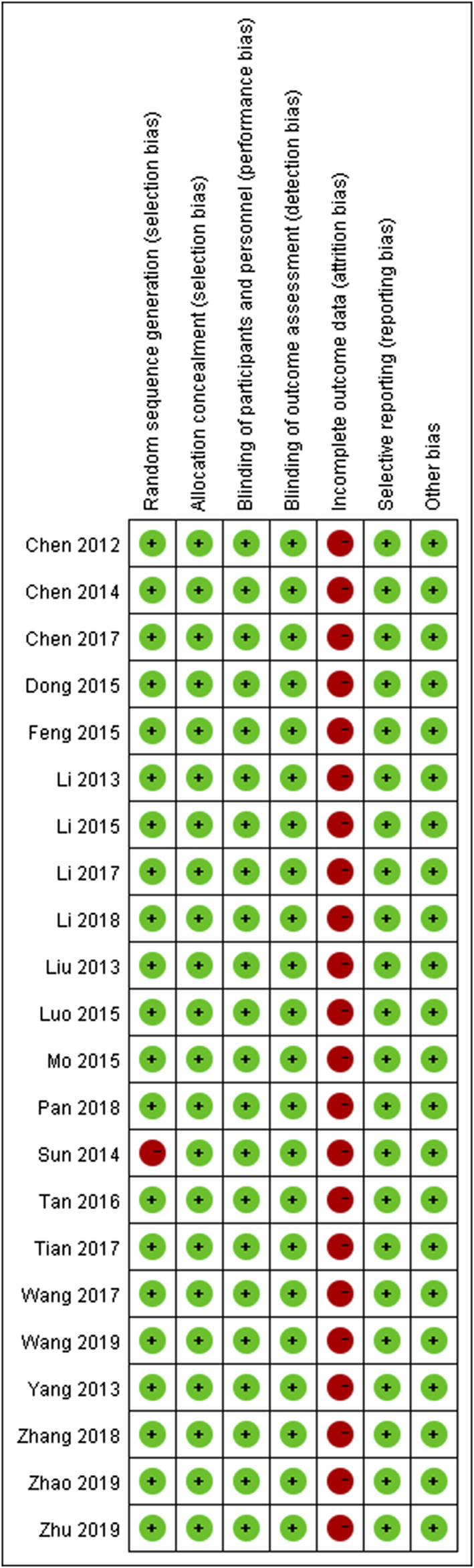
Risk of bias assessments within studies.

The majority of RCTs had a low risk of bias (Figure 2). The risk of other bias was the most common risk factor for quality assessment; the second risk factor was incomplete outcome data, given the unnecessary of reporting the whole results of a variety of parameters.

### Network Meta-Analysis Results

The clinical heterogeneity and methodological heterogeneity of the basic characteristics of the included studies were tested. The results showed that there was significant heterogeneity between studies, including Q test (*p* = 0.001), H value 2.124 [95% CI (1.548, 2.808)], I^2^ = 65.589%. However, after stratification of factors that may affect heterogeneity such as dosing frequency, duration, study year, and trial design, the heterogeneity test was performed, Q test (*p* > 0.05), and I^2^ = 24.245–49.479% indicated that there was no heterogeneity between studies within each layer. The results showed the comparison of each split node, direct comparison and indirect comparison was not statistically significant (*p* > 0.05), suggesting that there was no evidence that the network model was inconsistent.

### Pulmonary Artery Systolic Pressure (PASP)

**FIGURE 3:**
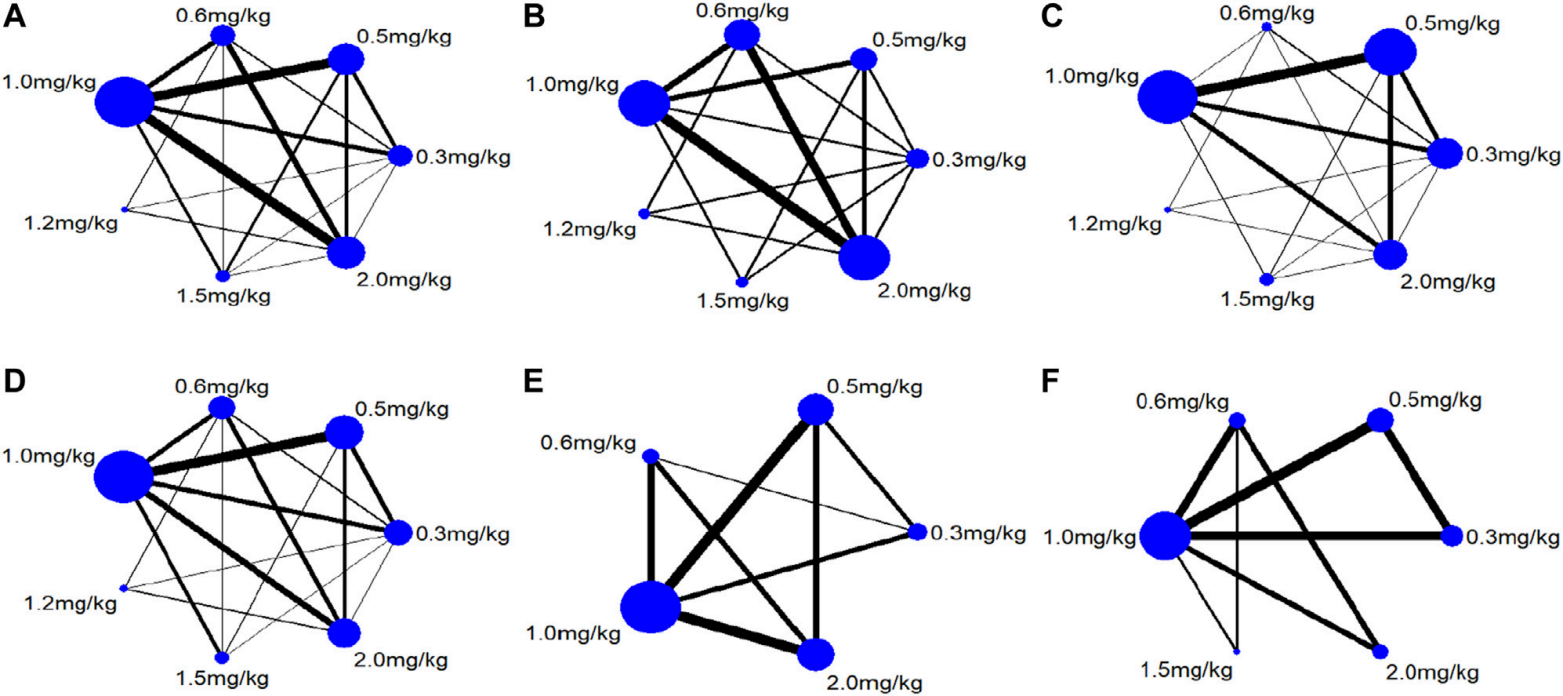
Relations diagram of different dose sildenafil for PPNN.

**FIGURE 4:**
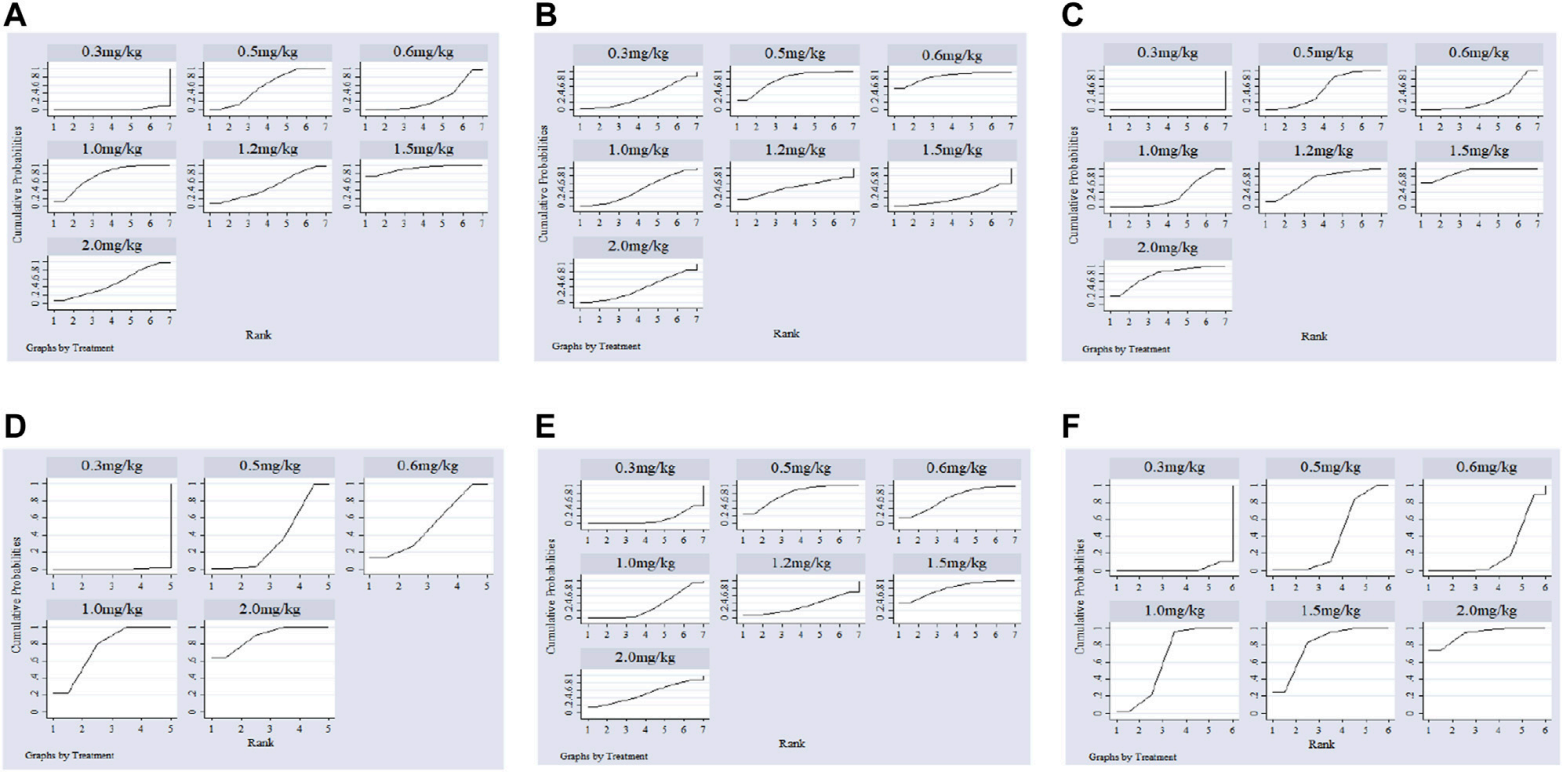
Index ranking chart of different doses of sildenafil for PPNN.

Twenty-two studies including 2,131 pediatric patients were included in the analysis. Sixteen studies were three-arm trials. Three studies each were two-arm trials and four-arm trials (Figure 3A). The node split method was used to test the consistency of the included studies. The test results showed that there was no significant difference between the direct comparison and indirect comparison (*p* > 0.05), except between 0.3 and 0.6 mg/kg (*p* = 0.046). The network meta-analysis was performed using an inconsistency model. The NMA revealed that, compared with 0.3, 0.6 and 1.5 mg/kg showed a significant decrease; compared with 0.6, 1.0 and 1.5 mg/kg showed a significant decrease in PASP (*p* < 0.05, Table 2). There was no significant difference between other treatments. Surface under the cumulative ranking analysis (SUCRA) showed that 1.5 mg/kg (92.0%, moderate certainty evidence) had the best effect on reducing pulmonary arterial pressure, followed by 1.0 mg/kg (74.6%), while 0.3 mg/kg (1.6%) had the lowest SUCRA value (Table 3; Figure 4A). NMA forest plot for different research combinations and combination effect sizes of PASP was presented in Supplementary Figure S1.

**TABLE 2:** Pairwise comparisons of the efficacy of different doses of sildenafil for PPHN.

Intervention	PASP	PaO_2_	SaO_2_	PaCO_2_	SBP	Clinical efficacy
0.3 mg/kg						
0.5 mg/kg	3.89 (0.00, 6740.91)	71.90 (2.90, 1779.91)^a^	43.68 (5.13, 371.60)^a^	0.25 (0.00, 70.58)	6.58 (0.41, 105.46)	1.62 (1.27, 2.05)^a^
0.6 mg/kg	0.00 (0.00, 0.00)^a^	2.97 (0.04, 215.48)	0.04 (0.00, 54.29)	0.00 (0.00, 0.00)^a^	18.82 (0.34, 1032.84)	1.13 (0.73, 1.704)
1.0 mg/kg	4.48 (0.00, 186059.44)	337.21 (16.37, 6948.57)^a^	0.04 (0.00, 54.29)	0.02 (0.00, 97.52)	1.42 (0.10, 19.31)	1.73 (1.36, 2.20)^a^
1.2 mg/kg	0.98 (0.00, 10423.26)	370.28 (0.20, 678234.24)	837.14 (40.71, 17214.65)^a^	0.00 (0.00, 0.13)^a^	2.09 (01, 567.77)	-
1.5 mg/kg	0.00 (0.00, 0.20)^a^	23813.84 (239.79, 2.27e+06)^a^	9206.58 (226.83, 373679.54)^a^	0.01 (0.00, 1.33)	0.46 (0.02, 11.08)	1.93 (1.30, 2.86)^a^
2.0 mg/kg	0.00 (0.00, 1.66)	5188.88 (100.32, 268375.68)^a^	-	0.01 (0.00, 1.35)	1.05 (0.05, 22.08)	2.20 (1.53, 3.16)^a^
0.5 mg/kg						
0.6 mg/kg	202.18 (0.2, 206104.25)	0.04 (0.00, 2.78)	19.16 (0.47, 778.83)	11.88 (0.12, 1190.11)	2.86 (0.05, 158.83)	0.70 (0.48, 1.03)
1.0 mg/kg	0.18 (0.01, 5.65)	4.69 (0.41, 53.86)	0.47 (0.05, 4.18)	5.34 (0.48, 59.16)	0.22 (0.03, 1.80)	1.07 (0.93, 1.24)
1.2 mg/kg	6.11 (0.00, 120300.74)	5.15 (0.00, 9495.04)	19.62 (3.75, 102.75)^a^	0.04 (0.00, 197.96)	0.32 (0.00, 106.82)	-
1.5 mg/kg	0.00 (0.00, 8.70)	331.20 (4.77, 22978.57)^a^	-	0.00 (0.00, 0.21)^a^	0.07 (0.00, 1.47)	1.19 (0.84, 1.69)
2.0 mg/kg	6.11 (0.00, 120300.97)	72.17 (2.52, 2068.98)^a^	-	0.01 (0.00, 72.82)	0.16 (0.01, 1.92)	1.36 (1.00, 1.85)
0.6 mg/kg						
1.0 mg/kg	0.00 (0.00, 0.91)^a^	113.60 (2.52, 5113.23)^a^	0.02 (0.00, 1.02)	0.45 (0.00, 45.34)	0.08 (0.00, 3.30)	1.53 (1.07, 2.18)^a^
1.2 mg/kg	0.03 (0.00, 1887.59)	124.74 (0.08, 190930.02)	1.02 (0.02, 59.29)	0.00 (0.00, 29.14)	0.11 (0.00, 37.83)	-
1.5 mg/kg	0.00 (0.00, 0.19)^a^	8022.2 (50.76, 1.27e+06)^a^	-	0.00 (0.00, 0.04)^a^	0.02 (0.00, 2.25)	1.70 (1.17, 2.48)^a^
2.0 mg/kg	0.03 (0.00, 1887.60)	1747.99 (26.35, 115974.34)^a^	-	0.00 (0.00, 10.72)	0.06 (0.00, 2.38)	1.95 (1.31, 2.89)^a^
1.0 mg/kg						
1.2 mg/kg	33.85 (0.00, 667976.51)	1.10 (0.00, 1763.18)	41.32 (2.72, 627.69)^a^	0.01 (0.00, 37.22)	1.47 (0.00, 448.57)	-
1.5 mg/kg	0.03 (0.00, 48.35)	-	-	0.00 (0.00, 0.04)^a^	0.32 (0.02, 5.96)	1.11 (0.81, 1.52)
2.0 mg/kg	33.85 (0.00, 667977.78)	-	-	0.00 (0.00, 13.69)	0.74 (0.07, 7.71)	1.27 (0.97, 1.66)
1.2 mg/kg						
1.5 mg/kg	0.00 (0.00, 71.12)	64.31 (0.02, 226547.83)	-	0.02 (0.00, 373.26)	0.22 (0.00, 102.55)	-
2.0 mg/kg	1.00 (0.00, 15904.36)	14.01 (0.01, 20316.32)	-	0.37 (0.00, 1117.36)	0.50 (0.00, 147.12)	-
1.5 mg/kg	-					
2.0 mg/kg	1274.11 (0.01, 15e)	0.22 (0.00, 27.08)	-	15.03 (0.00, 229183.77)	2.28 (0.07, 72.56)	1.14 (0.77, 1.69)

-, no data; PASP, pulmonary artery systolic pressure; SBP, systolic blood pressure; PaO2, partial pressure of oxygen; PaCO2, partial pressure of carbon dioxide; SaO2, arterial oxygen saturation.

a *p* < 0.05.

**TABLE 3:** Ranking of different doses of sildenafil for PPHN.

Intervention	PASP		PaO_2_		SaO_2_		PaCO_2_		SBP		Clinical efficacy	
	SUCRA (%)	Mean Rank	SUCRA (%)	Mean Rank	SUCRA (%)	Mean Rank	SUCRA (%)	Mean Rank	SUCRA (%)	Mean Rank	SUCRA (%)	Mean Rank
0.3 mg/kg	1.6	6.9	10.4	6.4	0.1	5.0	0.1	7.0	64.9	3.1	5.5	5.7
0.5 mg/kg	57.2	3.6	77.9	2.3	34.9	3.6	52.8	3.8	22.2	5.7	46.3	3.7
0.6 mg/kg	26.8	5.4	67.0	3.0	51.0	3.0	28.1	5.3	13.8	6.2	15.4	5.2
1.0 mg/kg	74.6	2.5	30.5	5.2	75.2	2.0	32.1	5.1	56.6	4.9	62.2	2.9
1.2 mg/kg	48.9	4.1	33.3	5.0	-	-	70.1	2.8	49.1	4.1	-	-
1.5 mg/kg	92.0	1.5	79.3	2.2	-	-	91.1	1.5	78.9	42.4	77.0	2.1
2.0 mg/kg	49.0	4.1	51.6	3.9	88.6	1.5	75.8	2.5	64.5	14.3	93.5	1.3

-, No data; PASP, pulmonary artery systolic pressure; SBP, systolic blood pressure; PaO2, partial pressure of oxygen; PaCO2, partial pressure of carbon dioxide; SaO2, arterial oxygen saturation; SUCRA, surface under the cumulative ranking.

### Systolic Blood Pressure (SBP)

Seven studies including 469 pediatric patients were included in the analysis. Among the RCTs, five studies were three-arm trials. Two studies were two-arm trials (Figure 3B). The node split method showed that there was no significant difference between the direct comparison and indirect comparison (*p* > 0.05). The network meta-analysis was performed using a consistency model. The NMA revealed that there was not a significant difference in reducing SBP (Table 2). SUCRA showed that 1.5 mg/kg (78.9%, moderate certainty evidence) had the best effect in reducing SBP, followed by 0.3 mg/kg (64.9%) (Table 3; Figure 4C). NMA forest plot for different research combinations and combination effect sizes of SBP was presented in Supplementary Figure S2.

### Partial Pressure of Carbon Dioxide (PaCO_2_)

Fourteen studies including 1,552 pediatric patients were included in the analysis. Nine studies were three-arm trials. Two studies were two-arm trials, and three studies were four-arm trials (Figure 3C). The node split method showed that there was no significant difference between the direct comparison and indirect comparison (*p* > 0.05), except between 0.3 and 1.0 mg/kg, 0.5 and 1.0 mg/kg, and 0.5 and 2 mg/kg (*p* < 0.05). The network meta-analysis was performed using an inconsistency model. The NMA revealed that 0.6 and 1.2 mg/kg were better than 0.3, and 1.5 mg/kg was better than 0.5, 0.6 and 1.0 mg/kg in reducing the partial pressure of carbon dioxide (*p* < 0.05, Table 2). SUCRA showed that 1.5 mg/kg (91.1%, moderate certainty evidence) had the best effect in reducing the partial pressure of carbon dioxide, followed by 2.0 mg/kg (75.8%), while 0.3 mg/kg (0.1%) had the lowest SUCRA value (Table 3; Figure 4C). NMA forest plots for different research combinations and combination effect sizes of PASP (Supplementary Figure S1). NMA forest plot for different research combinations and combination effect sizes of PaCO2 was presented in Supplementary Figure S3.

### Partial Pressure of Oxygen (PaO_2_)

Nineteen studies including 1771 pediatric patients were included in the analysis. Fifteen studies were three-arm trials. Two studies were two-arm trials, and two studies were four-arm trials (Figure 3D). The node split method showed that there was no significant difference between the direct comparison and indirect comparison (*p* > 0.05). The NMA was performed using a consistency model. The NMA revealed that 0.5, 1.0, 1.5, and 2 mg/kg were better than 0.3 mg/kg; 1.5 and 2.0 mg/kg were better than 0.5, 1.0, and 1.5 mg/kg; 2 mg/kg was better than 0.6 mg/kg; and 1.5 mg/kg was better than 1.0 mg/kg at increasing the partial pressure of oxygen. The differences were statistically significant (*p* < 0.05, Table 2). In contrast, no difference was found in the rest of the comparisons (*p* > 0.05, Table 3). SUCRA showed that 1.5 mg/kg (79.3%, moderate certainty evidence) had the best effect in increasing the arterial oxygen pressure, followed by 0.5 mg/kg (77.9%), while 0.3 mg/kg (10.4%) had the lowest SUCRA value (Table 3; Figure 4E). NMA forest plot for different research combinations and combination effect sizes of PaO2 was presented in Supplementary Figure S4.

### Arterial Oxygen Saturation (SaO_2_)

Thirteen studies including 1165 pediatric patients were included in the analysis. Among the RCTs, twelve studies were three-arm trials. One study was a two-arm trial (Figure 3E). The node split method showed that there was not a significant difference between the direct comparison and indirect comparison, except between 0.5 and 1.0 mg/kg and between 0.6 and 1.0 mg/kg (*p* < 0.05). The network meta-analysis was performed using an inconsistency model. The NMA revealed that 0.5, 1.2 and 1.5 mg/kg were better than 0.3 mg/kg; and 1.2 mg/kg was better than 0.5 and 1.0 mg/kg at increasing the arterial oxygen saturation (*p* < 0.05, Table 2). SUCRA showed that 2.0 mg/kg (88.6%, moderate certainty evidence), had the best effect in increasing the arterial oxygen saturation, followed by 1.0 mg/kg (75.2%), while 0.3 mg/kg (0.1%) had the lowest SUCRA value (Table 3; Figure 4D). NMA forest plot for different research combinations and combination effect sizes of PaO2 was presented in Supplementary Figure S5.

### Clinical Efficacy

Seven studies including 502 pediatric patients reported clinical effectiveness. Six studies were three-arm trials. One study was a two-arm trial, and one was a four-arm trial (Figure 3F). The node split method showed that there was no significant difference between the direct comparison and indirect comparison (*p* > 0.05). The network meta-analysis was performed using a consistency model. The NMA revealed that 0.5, 1.0, 1.5, and 2.0 mg/kg had higher clinical effectiveness than 0.3 mg/kg; and 1.0, 1.5, and 2.0 mg/kg had higher clinical effectiveness than 0.6 mg/kg (Table 2). SUCRA showed that 2.0 mg/kg (93.5%, low certainty of evidence) had the best effect in terms of clinical efficacy, followed by 1.5 mg/kg (77.0%), while 0.3 mg/kg (5.5%) had the lowest SUCRA value, as discussed earlier (Table 3; Figure 4F).

### Adverse Reactions

Four doses in nine articles described adverse reactions, including hypoglycemia (two cases), urticaria (three cases), tachycardia (two cases), and gastrointestinal bleeding (one case). Among them, the 1 mg/kg group and 2.0 mg/kg group experienced four types of adverse reactions. Hypotension did not occur at different doses of sildenafil among the newborns.

### Contribution Matrix

The influence of different direct comparisons on the results of NMA showed that the direct comparison of 1.2 and 2.0 mg/kg had the greatest influence on the results of the whole results (10.2%), followed by 0.5 and 1.0 mg/kg group (9.7%).

## Discussion

PPHN is an important cause of neonatal death and is considered a complex condition with a more or less unknown pathophysiology. PPHN can be primary (such as severe pulmonary hypoplasia) or can occur secondary to pulmonary parenchymal disease (such as meconium aspiration syndrome, surfactant deficiency, or alveolocapillary dysplasia). In addition, polycythemia, hypoglycemia, sepsis, maternal diabetes mellitus, cesarean section delivery, high maternal body mass index, and maternal use of aspirin, nonsteroidal anti-inflammatory drugs (NSAIDs) and serotonin reuptake inhibitors are among the factors associated with an increased risk of PPHN (Delaney and Cornfield, 2012). The degree of PPHN ranges from mild hypoxia with mild respiratory distress to severe hypoxemia with cardiopulmonary instability.

The purpose of PPHN treatment is to reduce pulmonary vascular resistance (PVR), maintain systemic blood pressure, correct right-to-left shunting and improve oxygenation (Abman et al., 2015; Hansmann et al., 2019). Treatment of PPHN includes mechanical ventilation, oxygen therapy, ECMO, surfactant, vasoactive drugs and so on (Abman et al., 2015; Hansmann et al., 2019). Vasoactive drugs include iNO, endothelin receptor antagonists (ETRAs), prostaglandins (PGE1 or PGI2), phosphodiesterase inhibitors, etc. Among them, iNO is the most investigated choice of treatment and the only approved specific pulmonary vasodilator for newborns (Tanriverdi et al., 2014). However, the high cost of iNO therapy remains an issue of serious concern in resource-limited settings. In addition, up to 30–40% of infants are nonresponsive to iNO (Goldman et al., 1996), and iNO does not reduce mortality or the length of hospitalization. In these settings, PDE inhibitors seem to fill the gap. Sildenafil is the most investigated non-iNO treatment.

Sildenafil is a selective phosphodiesterase 5 (PDE-5) inhibitor and a vasodilator specifically for the pulmonary vasculature that increases the intracellular cAMP and cGMP levels by inhibiting PDE, leading to vascular smooth muscle relaxation in the pulmonary vascular bed (Juliana and Abbad, 2005). Most of the current studies have confirmed the efficacy of sildenafil monotherapy or as an adjunct therapy to iNO for PPHN. The dose and indications have been derived from data from adults and older children. However, controversies about the dose regime, route and time intervals of sildenafil administration still need to be further investigated. In our study, we analyzed seven different doses of sildenafil for PPHN. The results showed that sildenafil administered every 6 h for 3 days within a dose range of 0.3–2.0 mg/kg by nasal feeding can significantly improve clinical symptoms such as dyspnea, cyanosis, blood oxygenation status and PASP in infants. However, the results show that there is a certain dose dependence, and the therapeutic effect becomes more and more significant as the dose increases. Consequently, in terms of reducing PASP, the effect of reducing PASP was more obvious with increasing doses in the range of 1.5 mg/kg, while 2.0 mg/kg showed no difference, indicating that increasing the dose could not further reduce PASP. Similarly, 1.5 mg/kg had the most significant effect in increasing the PaO2 and reducing the PaCO2. Our found that some infants have increased or decreased SBP after treatment, but it is not statistically significant compared to before sildenafil treatment. But the cause of the increase in SBP is unclear. All the RCTs in this study used different doses of sildenafil to compare the clinical efficacy within 3 days, indicating that short-term high-dose sildenafil is more effective than low-dose sildenafil, but the long-term efficacy still needs further research and observation. Barst RJ et al. (Barst et al., 2014) have shown that higher doses of sildenafil can increase the mortality of children with pulmonary arterial hypertension. Similarly, as the dose increased, the effect of increasing the arterial blood oxygen pressure and reducing the partial pressure of carbon dioxide were better, and the SUCRA ranking showed that 1.5 mg/kg had the best effect. In terms of increasing arterial oxygen saturation and clinical effectiveness, the SUCRA ranking showed a better effect at 2.0 mg/kg.

Despite the fact that we have limited knowledge of sildenafil safety in PPHN, previous studies have reported that the most frequently reported adverse reactions (AEs) were priapism, facial flushing, headaches, nasal stuffiness, irritability, diarrhea, vomiting, cough, abnormal vision and upper respiratory tract infection (Beghetti et al., 2017). The AEs increase as the dose increases in adult and children aged 1–17 yr (Rubin et al., 2011), and the majority of AEs were of mild or moderate intensity. Sildenafil coadministered with iNO might reduce the systemic arterial pressure (SAP), while sildenafil alone did not result in such an effect (Beghetti et al., 2017). AEs, such as urticaria and tachycardia were observed in the present work and were of mild to moderate severity, while hypotension did not occur at different doses of sildenafil. This finding demonstrates that sildenafil monotherapy appears to be safe and well tolerated. However, it is necessary to carry out additional studies to assess the side effects in neonates (Ahsman et al., 2010). previously investigated the pharmacokinetics of repeated administration of sildenafil in 11 neonates ranged in postnatal days from 2 to 121 days with pulmonary hypertension. The patients had received sildenafil (0.5 mg/kg) three or four times daily in the intensive care unit (ICU). Based on their research, the pharmacokinetic variability of sildenafil is quite big. The median AUC24 (sildenafil + desmethylsildenafil) was 3,935 ng/h/ml (range 625–13,579 ng/h/ml). However, it is still necessary to carefully adjust the dose.

## Conclusion

In conclusion, sildenafil is effective and safe for decreasing the PASP in newborns with PPHN. Our research proves that 1.5 mg/kg has the best effect in reducing pulmonary arterial pressure and the partial pressure of carbon dioxide and increasing the arterial blood oxygen pressure without severe adverse effects. However, the limitations of this study were as follows: *1*) This study only included literature published in Chinese; other studies in English were not found; *2*) the 22 studies included were all RCTs, but the quality of the literature was not high; one study did not describe the method of random sequence generation and allocation concealment; *3*) the number of included studies was small, and the results of the publication bias analysis may not be reliable; *4*) the follow-up time of the included studies was 1–3 days, which is short, suggesting that follow-up studies need to increase the follow-up time to obtain safety data; and *5*) in the study, infants were treated the brand name drug and generic drugs. The brand name drug and generic drug of sildenafil have bioequivalence, but the clinical equivalence of the brand name drug and generic drugs needs further study. Therefore, large RCTs are needed before advocating the clinical use of this agent in neonates with PPHN. Furthermore, the dose route and time intervals of sildenafil administration still need to be further elucidated.

## Data Availability

Publicly available datasets were analyzed in this study. The original contributions presented in the study are included in the article/Supplementary Material, further inquiries can be directed to the corresponding author.

## Supplementary Material

Supplementary Figure 1 Network forest plot of PASP Pulmonary Artery Systolic Pressure (PASP).

Supplementary Figure 2 Network forest plot of SBP Systolic Blood Pressure (SBP).

Supplementary Figure 3 Network forest plot of PaCO2 Partial Pressure of Carbon Dioxide (PaCO2).

Supplementary Figure 4 Network forest plot of PaO2 Partial Pressure of Oxygen (PaO2).

Supplementary Figure 5 Network forest plot of SaO2 Arterial Oxygen Saturation (SaO2).
