# Supplementary figures and images for: Clinical Efficacy and Safety of Different Doses of Sildenafil in the Treatment of Persistent Pulmonary Hypertension of the Newborn: A Network Meta-analysis
Source: Front Pharmacol. 2021 Sep 24;12:697287. doi: 10.3389/fphar.2021.697287 (PMC8497971; doi:10.3389/fphar.2021.697287)

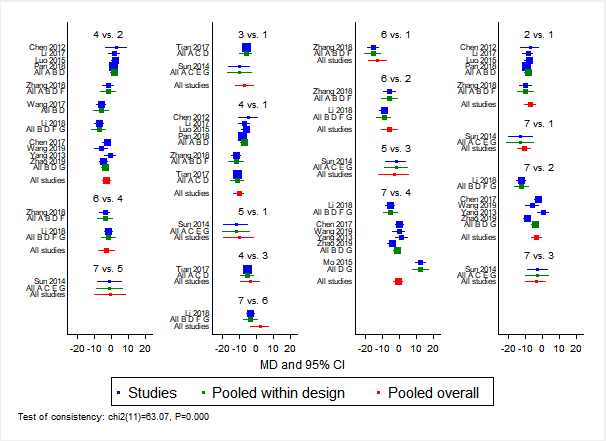

Supplement: Supplementary file 1 [file Image3.TIF]

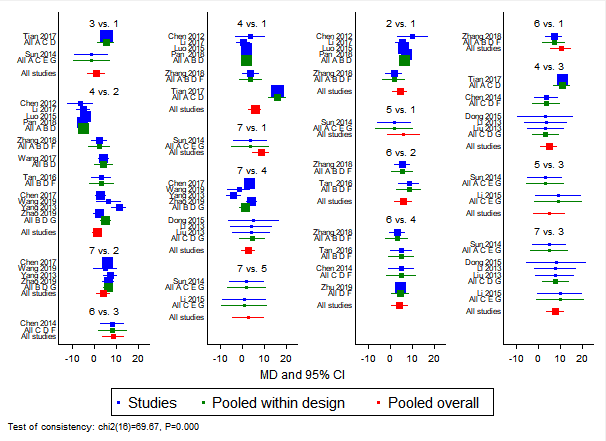

Supplement: Supplementary file 2 [file Image4.TIF]

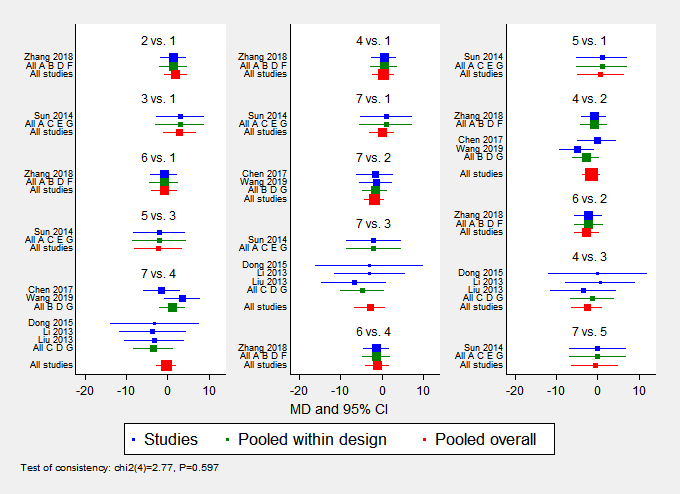

Supplement: Supplementary file 3 [file Image2.TIF]

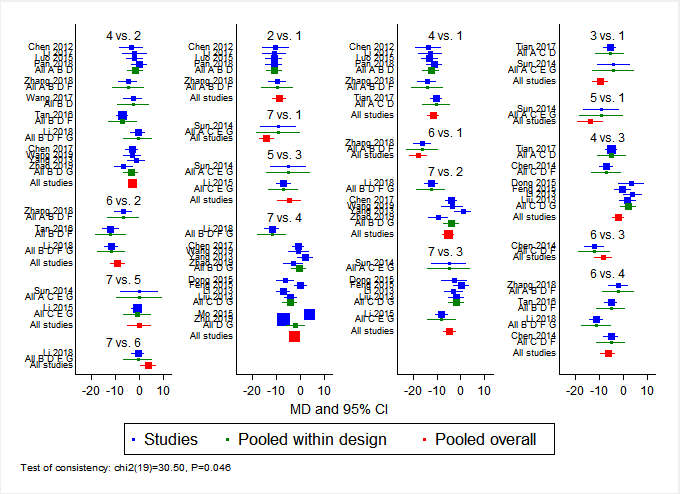

Supplement: Supplementary file 4 [file Image1.TIF]

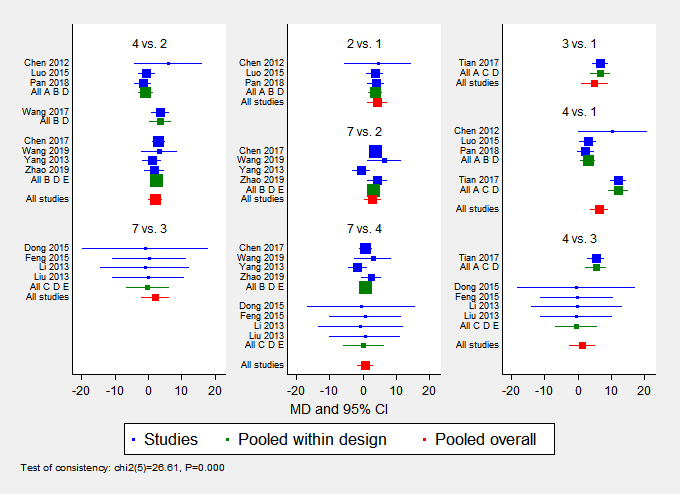

Supplement: Supplementary file 5 [file Image5.TIF]
